# Impact of Bean Consumption on Nutritional Outcomes amongst Adolescents

**DOI:** 10.3390/nu12041083

**Published:** 2020-04-14

**Authors:** Ana Paula Fernandes Gomes, Ana Carolina Carioca da Costa, Edna Massae Yokoo, Vania de Matos Fonseca

**Affiliations:** 1 Postgraduate Program in Child and Women’s Health, National Institute of Women’s, Child and Adolescent Health Fernandes Figueira, Oswaldo Cruz Foundation, 20021-140 Rio de Janeiro, Brazil; 2 Department of Fundamental Nutrition, Federal University of the State of Rio de Janeiro, 22290-240 Rio de Janeiro, Brazil; 3 National Institute of Women’s, Child and Adolescent Health Fernandes Figueira, Oswaldo Cruz Foundation, 20021-140 Rio de Janeiro, Brazil; ana.costa@iff.fiocruz.br (A.C.C.d.C.); vaniamf@iff.fiocruz.br (V.d.M.F.); 4 Department of Epidemiology and Biostatistics, Fluminense Federal University, 24220-000 Niterói, Brazil; eyokoo@gmail.com

**Keywords:** adolescent, food behaviour, food consumption, nutritional status, Fabaceae, obesity

## Abstract

Brazilian adolescents have undergone a noteworthy nutritional epidemiological transition. There is an increase in the prevalence of overweight and high consumption of ultra-processed foods in parallel with patterns of traditional meals that include beans. This study analyzed associations between bean consumption in the diet of adolescents and nutrition outcomes. Multiple regression analysis showed a significant reduction in body mass index (BMI), body fat percentage (%BF) and LDL-cholesterol (LDL-c) values among those with bean consumption equal to or greater than five times a week. Adolescents who had lunch outside the home and those who did not have the habit of having lunch showed a significantly higher BMI. There was an increase in the %BF among married adolescents and those who did not have lunch. There was a reduction of LDL-c among those with intermediate per capita income and those who consumed processed juice less than 5 times a week, and an increase among those who did not have breakfast. There were significant interactions between sexual maturation, energy consumption, physical activity and energy consumption. Thus, in the context of this study, the presence of beans in the diet, at frequencies equal to or greater than five times a week, can be considered a proxy for healthy eating.

## 1. Introduction

According to The Global Health Observatory of the World Health Organization, the prevalence of overweight among Brazilian adolescents jumped from 6.8% to 26.2% between 1975 and 2016. This report ratified prior findings from a previous Family Budget Survey (Pesquisa de Orçamento Familiar, POF) showing a constant increase in the prevalence of overweight and obesity in the adolescent population conducted between 1974 and 2009 (National Family Expenditure Survey 1974–1975; National Health and Nutrition Survey 1989; POF 2002–2003 and 2008–2009) [1,2].

Excess weight in adolescents triggers emotional, social and economic consequences for them, for the family and for society. There is a greater probability of becoming an overweight adult and being at a higher risk of developing chronic diseases. For females in particular, it can also mean greater risk of affecting other life stages, such as pregnancy, lactation and the mother–child relationship [3,4,5].

Several factors can interfere with adolescent food consumption: political and commercial factors, income, social and family norms, media influence, longer periods away from home and physical activity, among others [3,6,7,8]. Regardless of the consecutive analysis indicating a change in diet patterns and an increase in consumption of ultra-processed foods that are replacing home cooked meals, the Brazilian population, adolescents included, regularly consume beans [2,7,9,10,11,12,13,14,15,16]. Some studies have shown that adolescents opt for snacks and commercial foods instead of regular meals, leading to high consumption of sodium, simple sugars and saturated fats, and low consumption of calcium, folate, iron and fibers. Other studies have shown the opposite, with a predilection of this group for beans [10,11,13,14,17,18].

In view of the indubitable role that beans play in the Brazilian diet and the current epidemiological and nutritional transition observed among adolescents, this study aims to examine the associations between consumption of beans in the diet of adolescents and nutritional outcomes, in particular body mass index (BMI), body fat percentage (%BF) and LDL-cholesterol (LDL-c). Our hypothesis is that adolescents of both genders presenting with healthier anthropometric and biochemical indices partake in higher frequencies of bean consumption.

## 2. Methods

### 2.1. Study Design

This cross-sectional observational study obtained data from the Camelia (Cardio-metabolic-renal and family) project carried out by the Fluminense Federal University (UFF), in the city of Niterói, Rio de Janeiro, Brazil, during 2006 and 2007, with adults assisted by the Family Doctors Program (FDP), as well as their spouses and biological children aged between 12 and 30 years.

Eligible participants in the present analysis were all children of adult members of Camelia aged between 12 and 19 years. Of a total of 362 families, 185 had children in this age group, totaling 247 adolescents, but we included in the analysis only those with food consumption data. Two hundred and thirty-two (232) were eligible for the study, ninety-four percent (94.0%) of all adolescents. The participants answered a standardized questionnaire, covering demographic, socioeconomic, concomitant and lifestyle characteristics. By means of a medical consultation we assessed blood pressure and collected blood and urine samples as well as anthropometric measurements and nutritional parameters.

### 2.2. Demographic, Socioeconomic and Lifestyle Characteristics

Age was recorded in completed full years at the time of the interview. Skin color (white, colored or black) was self-reported by the adolescents. Schooling refers to the highest grade of attendance, completed or not. Family income was calculated using the information provided by guardians on total earnings of all family members in the month prior to the survey. This figure was divided by the total number of people in the household giving the per capita income in Brazilian Reais.

The physical activity questionnaire contained questions about exercise performed in the last 15 days, recorded by frequency per week and duration of sessions [19]. Information on time spent watching television and videos was also collected (sedentary activities). Adolescents were considered active when they had moderate or intense physical activity for at least 60 min and 5 or more days per week (300 min per week) [20].

They were classified as smokers when they answered positively to the question: “Are you or have you ever been a smoker, that is, have you smoked, during your life, at least 100 cigarettes (five cigarette packs)?” [21].

For evaluation of alcohol intake answers were considered as YES—“drink at least once a week”; and NO—“I’ve never taken it,” “I stopped drinking,” “rarely (<1 time over 3 months)” and “occasionally (<1 time a month)”.

### 2.3. Biochemical Characteristics and Blood Pressure

Biochemical measurements of blood and urine samples were collected after a 12 h fast and were analyzed at the Vizela Laboratory of the Municipal Health Foundation and at the Antônio Pedro University Hospital (HUAP) of UFF. Biochemical evaluations performed using the Wiener^®^ Selectra equipment were: glucose (Gli) by hexoquinase method; insulin by chemiluminescence; total cholesterol (TC) and triglycerides (TG) by enzymatic kinetics; and HDL-cholesterol (HDL-c) and LDL-c by enzymatic colorimetric assay. HbA1c was measured by immunoturbidimetry using a Labmax 240 equipment from Labtest. Sample collection and biochemical analyses took place on the same day. Blood pressure was measured three times with a Pro Check^®^ digital manometer and a standardized oscillometric blood pressure device (OmronHealthcare Incorporated, Lake Forest, USA), allowing for calculation of the mean value between the second and third measurements [22]. When the difference was higher than 5 mm Hg between measurements, a new measurement was carried out.

### 2.4. Anthropometric Characteristics

Anthropometric measurements were performed following standardization of Lohman and collaborators [23]. Weight in kg was assessed once with adolescents wearing as little clothing as possible, using the digital scale of the Filizola^®^ model PL18 (Filizola S/A Industry, São Paulo, Brazil), 150 kg capacity and 100 g accuracy. For height measurements we used a digital stadiometer (Kirchner & Wilhelm, Medizintechnik^®^, Asperg, Germany), with 1.0 cm precision. From weight and height, we calculated and ranked the body mass index, BMI=weight/height^2^ [24]. Waist and abdominal circumferences were measured twice by the same person, having the participant exhaling, using a 200 cm long non-stretching measuring tape, with 0.1 cm precision. The intra-measurement mean was calculated to a maximum variation of 1 cm. The procedure was repeated if the mean variation was above such a limit. The measurements of abdominal and waist circumference were taken at the level of the left iliac crest and at the midpoint distance between the iliac crest and the last costal edge, respectively. The circumference of the hip was measured at the larger perimeter, passing through the buttocks, with the individuals in upright position, arms along the body and feet together. The waist–hip ratio (WHR) and waist–height ratio (WER) were calculated from these measurements. The tricipital skinfold thickness (TST) was measured in a standardized location and the %BF was also measured by bio impedance [25]. Age of menarche and axillary pilosity were used to determine the biological maturation phase [26]. To determine biological maturation, we asked age of menarche for girls (yes/no), and for boys we considered the axillaries pilosity (yes/no) observed during clinical examination by trained nurses and medical doctors of the research project.

### 2.5. Eating Habits

The adolescents’ food consumption was assessed using the Food Frequency Semiquantitative Questionnaire (FFSQ) [27,28]. The frequency of consumption reported for each of the food items included in the FFSQ was then converted into daily frequency. To avoid overestimation (reports of daily frequency “2 or more times a day” and “5 or more times a week”), the calculation of daily frequency was differentiated, with the lowest possible daily frequency being considered.

The frequency of daily consumption for each food item was multiplied by the weight of the homemade measuring standard used in the FFSQ to obtain the intake in gm or ml per day. The NutWin Nutrition Support Program was used to estimate the daily energy consumption (Kcal) and nutritional composition, using the United States Department of Agriculture‘s food composition table as reference [29,30].

Food intake was calculated from our data on consumption frequencies and by groups of food: fruits; vegetables [31]; fruits and vegetables (FV) [32]; soft drinks and processed juice [11]. Bean consumption levels were classified into three categories: up to 4 times per week; 5 to 6 times per week to once daily; and twice or more daily. The number of dessert spoons of sugar added to drinks was categorized into: 1 to 2 dessert spoons and ≥3 dessert spoons (“3 to 4” and “5 or more”). The location of meals were categorized as: at home; out of the house (“at school” or “other”); and does not have it (“I don’t have breakfast” or “I don’t have lunch” or “I don’t have dinner”).

Food consumption was also classified according to the extent and purpose of the industrial level of processing defining each group, the NOVA classification. Group 1—unprocessed or minimally processed foods; Group 2—processed culinary ingredients; Group 3—processed foods; Group 4—ultra-processed food and drink products [33]. We merged Groups 1 and 2 into a single group called “culinary preparations”.

### 2.6. Statistical Analysis

The continuous variables are presented in median and percentile values (25 and 75), while categorical variables are shown in absolute frequencies and percentages. Bivariate analyses were performed in order to verify the association between anthropometric and biochemical profiles of adolescents and the frequency of bean consumption. Mann–Whitney and Kruskal–Wallis tests were used to evaluate the relation of nutritional status indices to two and three consumption groups, respectively. To estimate factors associated with nutritional status, linear regression models were used, with BMI, %BF and LDL-c as outcomes, and frequency of bean consumption as the main exposure variable in addition to gender, age, maturational stage, caloric intake and level of physical activity of adolescents as confounding factors. To verify the existence of multicollinearity, we used the variance inflation factor (VIF). Univariate regression analyses were performed and the variables that presented *p*-value below 0.20 were initially included in the multiple regression model. Then, a stepwise regression was performed, maintaining bean consumption and confounding factors fixed in the model. Interactions were also tested for each outcome variable. The level of significance used was 5%. The adequacy of the models was verified through the residual analysis. We used SPSS (version 22) and R (version 3.6.1) software for data analysis.

### 2.7. Ethical Considerations

The Cardio-metabolic-renal and family Project was conducted according to the principles established in Resolution 196/96 and the Declaration of Helsinki (review of 2000—Scotland) and was approved by the Research Ethics Committee of the Faculty of Medicine of the UFF/HUAP (CEP CMM/HUAP N°. 220/05).

## 3. Results

The general characteristics of the 232 adolescents studied are shown in Table 1. Of the total, 52.2% were female, 51.1% brown and more than 50% belonged to the lowest income stratum. The median age was 15 years and 145 of them (62.5%) showed advanced pubertal staging indicators (70.2% of the girls reported menarche and 54.1% of boys showed axillary pilosity). They had schooling above the 5th grade of elementary school (85.7%), were single (98.3%), only studied (89.1%), did not smoke (97.4%), did not drink alcohol (90.2%) and 75.7% reported physical activity less than 300 min per week (85.6% of girls and 64.6% of boys).

Table 2 shows the results of anthropometric characteristics and eating habits. The median BMI was 20.68 Kg/m^2^ (21.42 Kg/m^2^ in girls and 19.97 Kg/m^2^ in boys); the majority had adequate nutrition (67.5%) and overweight was present in 30.3% of the sample (girls 35.5% and boys 24.5%).

Regarding eating habits, 92.6% reported consumption of fruit and 83.1% of vegetables smaller than 3 times a day; 86.1% consumed FV less than 5 times a day. Regarding the consumption of sugar and sugary drinks, 35.9% reported consuming 3 or more dessert spoons of sugar daily. They also consumed soft drinks (73.7%) and processed juices (67.1%) less than 5 times a week. Finally, as for the location of meals, they predominantly had breakfast (81.3%), lunch (89.3%) and dinner (92.2%) at home.

Table 3 shows anthropometric and clinical outcomes of adolescents, stratified by the frequency of bean consumption. There were statistically significant differences in BMI, %BF, WER, TST, TC, LDL-c, Non HDL-c between the lowest (up to 4 times a week) and the highest frequency (2 or more times a day), and also in Gli, CT, LDL-c and not HDL-c between consumption frequencies 5 to 6 times up to 1 time a day and 2 or more times a day. All results were higher in the frequencies of bean consumption up to 4 times a week, except for glucose.

**Figure 1:**
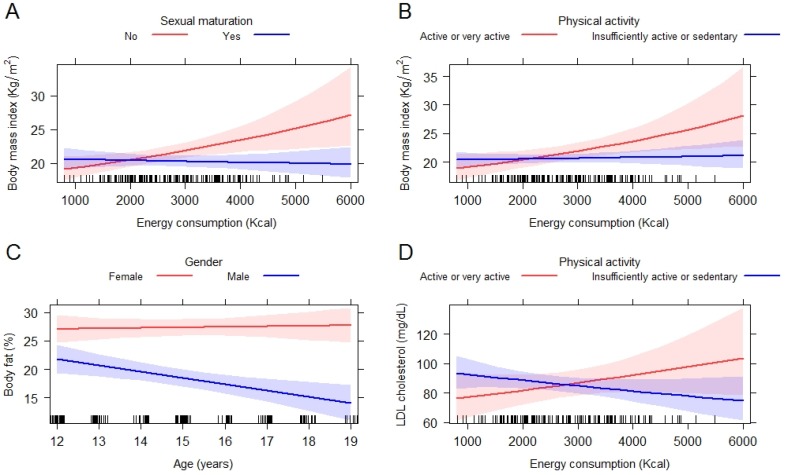
Interactions between (**A**) energy consumption and sexual maturation; (**B**) energy consumption and physical activity; (**C**) age and gender; (**D**) energy consumption and physical activity for nutritional outcomes of adolescents. Niterói—RJ, 2006–2007.

Table 4 shows the results of the multiple regression analysis for nutritional and biochemical status outcomes. Variables for the multiple regression model were: skin color, schooling, marital status, occupation, per capita income, smoker, alcohol consumption, sedentary activities, physical activity, fruits, vegetables, FV, added sugar to daily beverages, soft drinks, processed fruit juices, breakfast place, lunch place and dining place. There is a significant reduction in BMI values among adolescents who declared consuming beans 2 or more times a day, compared to those with consumption up to 4 times in the week. Compared to adolescents who had lunch at home, a significantly higher BMI was found among those who had lunch away from home and among those who did not have the habit of having lunch. There are also significant interactions between sexual maturation and energy consumption as well as between physical activity and energy consumption (Table 4). Figure 1A shows that female adolescents who already had menarche and boys with axillary pilosity tend to maintain BMI regardless of increased energy consumption. This did not occur among adolescents without these pubertal maturation traits. Among these, BMI tends to be higher as energy consumption increases. Active or very active adolescents also tend to have a higher BMI, once there is an increase in energy consumption (Figure 1B). Table 5 shows that the adolescents with higher energy consumption are also frequent consumers of processed and ultra-processed foods, combined with low consumption of FV and unprocessed or minimally processed foods.

In reference to %BF, this index is significantly lower among those who consume beans 5 to 6 times a week up to 1 time a day (*p* = 0.029) and 2 or more times a day (*p* = 0.024). Furthermore, %BF is higher among married adolescents (*p* = 0.034) and among those who do not take lunch (*p* = 0.004). For the interaction between gender and age, as male adolescents get older %BF tends to decrease which is not true among girls (*p* = 0.012) (Figure 1C).

For LDL-c the levels are lower among adolescents who consume beans 5 to 6 times a week up to once a day (*p* = 0.012) and 2 or more times a day (*p* = 0.007). Significantly lower LDL-c cholesterol levels were also verified among those with per capita income between R$200.00 and R$400.00 (*p* = 0.042), when compared to those with lower income as well as those whose consumption of processed juice was less than 5 times per week (*p* = 0.040). There is an increase of these levels among those who do not take breakfast (*p* = 0.009). Similarly to BMI, there is interaction between physical activity and energy consumption (Figure 1D). Increased energy consumption leads to increased LDL-c among physically active or very active adolescents, and not among inactive adolescents. Regarding BMI and LDL-c, the adoption of a healthy diet with higher amounts of culinary preparations, fruits and vegetables and smaller amounts of processed and ultra-processed foods also influences such a relationship (Table 5).

The residual analysis of the proposed regression models did not reveal any evidence of violation of the criteria for adequacy of the models (data not shown).

## 4. Discussion

The results of the present study ratify the association of better nutritional status with higher consumption of beans among adolescents. It also corroborates the idea that high consumption of fruits, legumes (beans) and vegetables may be considered markers of a healthy diet [11,13,33].

National, household or school-based studies of adolescents between 10 and 19 years of age, in the same region of the country (southeast region), showed a prevalence of overweight ranging from 20% and 27%, mainly among males [2,7,11]. In the present study, the prevalence of overweight was higher and occurred mainly in females. Such differences may be related to the fact that the adolescents studied here were selected in health units, as opposed to home or school samples, making this study peculiar. The prevalence of overweight observed here is quite worrying. These adolescents are at risk for several disorders, since obesity is positively associated with an increase in visceral fat deposits and a higher rate of metabolic complications and cardiovascular diseases [17,34,35].

In the case of eating habits of adolescents in the region, the results of the Study of Cardiovascular Risks in Adolescents (ERICA) indicated beans as the second most consumed food [13]. In the National School Health Survey (PENSE), the weekly consumption of beans equal to or greater than five days reached 60.7%, lower than what it is observed in this study, where a frequency equal to or greater than 5 times a week reached 85.4% [11].

In the present study, the multiple regression analysis showed that consumption of beans equal to or greater than five times a week was significantly important for the reduction of the three selected outcomes: BMI, %BF and LDL-c. Other authors reported on the frequency of consumption of legumes and the nutritional status of adolescents. In Ethiopia, a study of female adolescents aged between 15 and 19 years showed a high prevalence of stunting and wasting; their diets were composed predominantly of cereals, and the intake of fruits, animal products and legumes were particularly low [36].

In Chile, thinness (51.7%) and normal weight (47.0%) were more prevalent in groups that consumed legumes 2 to 5 days a week and excess weight was measured among those who consumed legumes less than once a week [37].

Eating meals, in particular lunch and breakfast, also proved to be important for the selected outcomes. The habit of having breakfast was predominant in our sample, as well as in other studies. Equally, it was related to improved health, depicted in the present study by the decrease of LDL-c [37,38,39]. The association between the frequency of daily meals and nutritional outcomes showed that having less than 4 meals per day was directly linked with increased BMI and LDL-c [8]. Skipping meals can expose the adolescent not only to inadequate food replacements, but may also contribute to faltering satiety, causing greater intake in the next meal and giving rise to glycaemic and insulin peaks as a response to greater secretion of lipoproteins [39,40].

Lunch was the most frequent meal, also pointed out by another study [37]. Declining lunch, a meal very likely to include beans, was associated with worsening adolescents’ nutritional status. Particularly with regard to BMI, it may be related to food choices outside the home environment.

POF data (2017–2018) showed a national increase of 8.7% in the weight of food expenditure if away from home, compared to POF 2002–2003 [41]. The National Consumers’ Price Index has registered regular increases and has been strongly affected by the Food Expenses group, that is, by items that make up culinary preparations. In addition, the Index confirmed increase in eating away from home and greater increases of the culinary preparation products price than in snacks [42]. Such increases allow us to reflect on the type of choice most likely to be made by low-income teenagers when they have lunch outside the home, since the costs of traditional meals tend to be higher than snacks. A study that evaluated eating patterns outside the home showed that adolescents were the group with the lowest adherence to a “traditional meal” that included beans, and the highest adherence to an “ultra-processed food” pattern [9].

The intermediate per capita income was also associated with a decrease in LDL-c compared to a lower income, but not compared to higher. Income is one of the factors determining diversity of the diet and the number of meals; particularly, it has an effect on the intake of fruits, vegetables and beans and also on the increase in the consumption of ultra-processed foods, aggravating the lipid profile and chronic non-communicable diseases [8,14,33]. The time-based analysis of four national household surveys (POF 1987–1988, POF 1995–1996, POF 2002–2003 and POF 2008–2009) showed an increase in the participation of ready-to-eat products across all income strata, but especially among those with lower income [2].

As with income, consumption of processed juice fewer than 5 times weekly was associated with a decrease in LDL-c. A study carried out with children and adolescents found a negative association between those who consumed sugary drinks in frequencies greater than two servings a day and the consumption of vegetables, fruits, beans, whole grains, dairy products, and seafood and vegetable proteins [43]. Diets rich in carbohydrates with fast absorption and low in fiber content have been associated with the onset of obesity, dyslipidaemias and diabetes, among other diseases [44].

Results on the occurrence of menarche and the axillary pilosity showed that BMI tends to be maintained even with increased energy consumption. This can be attributed to the fact that when adolescents report the occurrence of menarche, there is an indication that they have gone through the maximum height velocity and completed most of the growth process [26].

Regarding the % BF, we observed that it tends to decrease as boys get older, but does not decrease among girls. Changes in adipose tissue and its distribution during adolescence are influenced by age, gender, sexual maturation, human biological variability and genetic and environmental factors [4,26,45]. Thus, the small differences between genders, already present during childhood, increase over time including the fat-free mass. Girls tend to accumulate more adipose tissue than boys over time [4,45].

Another perceived interaction was an increase in BMI and LDL-c among adolescents who reported 300 or more minutes per week of activity, due to the increase in energy consumption. Physically active teenagers tend to have higher food intake, but not necessarily a healthy diet. Our results show that they consume more ultra-processed foods and fewer FV and culinary preparations, which can lead to increases in BMI and LDL-c [19]. In this regard, our results disagree with studies that showed an association between sedentary or insufficiently active adolescents and higher prevalence of daily consumption of ultra-processed foods or lower intake of unprocessed or minimally processed foods, as the study pointed to a higher consumption of ultra-processed foods among eutrophic adolescents, compared to those with excess weight [14,46]. It should be noted, however, that body mass index does not differentiate fat and lean mass.

It is of concern that the highest prevalence of overweight was among female adolescents (61.4% versus 38.6% male), combined with a higher prevalence of physical inactivity (60.2% among females versus 39.8% among boys) plus lower frequency bean consumption (up to 4 times a week) (70.6% versus 26.4%), especially when we take into account the changes in body composition that occur in females during puberty. Such conditions make us think that girls may be less protected in relation to boys against health problems, which raises the need for future studies.

One of the limitations of this study is the fact that this is part of a larger research project in which the variables studied had already been established, and it is not possible to change their form of presentation or influence their quality, coverage or condition. In addition, the results of food consumption were obtained from the Food Frequency Questionnaire, an instrument that can overestimate consumption, in addition to the possibility of losing other information. Another limitation is the fact that the study is of a cross-sectional nature and, therefore, one cannot assume cause and effect of diet on nutritional status.

## 5. Conclusions

Our findings showed that dietary patterns with higher frequencies of bean consumption were associated with lower BMI values and lower values of %BF and LDL-c. The presence of beans in the diet at frequencies equal to or greater than five times per week can be considered a proxy for healthy eating. In addition, having lunch away from home or skipping lunch, along with being married, were associated with increases in BMI and %BF. Moreover, the omission of breakfast and consumption of processed juices, at frequencies equal to or greater than 5 times per week, were associated with increases in LDL-c, and the intermediate per capita incomes with a decrease in LDL-c.

## Figures and Tables

**Table 1:** General characteristics of adolescents. Niterói—RJ, 2006–2007.

	Median (P25–75)	n (%)
**Age (years)**	15 (13–17)	
**Gender**		
Female		121 (52.2)
Male		111 (47.8)
**Skin color**		
Black		55 (24.0)
Colored		117 (51.1)
White		57 (24.9)
**Menarche/axillary pilosity**		
Yes		145 (62.5)
No		87 (37.5)
**Schooling**		
Basic literacy (up to 2nd grade)		17 (7.4)
Up to 4th grade		16 (6.9)
5th to 8th grade		143 (61.9)
1st year or more, secondary school		55 (23.8)
**Marital status**		
Single		228 (98.3)
Married or with companion		4 (1.7)
**Occupation**		
Studying		204 (89.1)
Working		13 (5.7)
Studying and working		6 (2.6)
Unemployed/homemaker		6 (2.6)
**Per capita income**		
Up to R$200.00		130 (57.8)
>R$200.00 up to R$400.00		70 (31.1)
>R$400.00		25 (11.1)
**Smoker**		
Yes		6 (2.6)
No		224 (97.4)
**Alcohol consumption**		
Yes		22 (9.8)
No		203 (90.2)
**Sedentary activities**		
<2 h/day		30 (13.8)
≥2 h/day		188 (86.2)
**Physical activity**		
<300 min/week		128 (75.7)
≥300 min/week		41 (24.3)

**Table 2:** Eating habits among adolescents. Niterói—RJ, 2006–2007.

	n	%
Fruits (n = 231)		
≥3× day	17	7.4
<3× day	214	92.6
Vegetables (n = 231)		
≥3× day	39	16.9
<3× day	192	83.1
FV (n = 231)		
≥5× day	32	13.9
<5× day	199	86.1
Beans (n = 232)		
Up to 4× per week	34	14.7
5 a 6× per week up to 1 daily	50	21.6
2 or plus× daily	148	63.8
Added sugar to daily beverages (n = 220)		
1–2 dessert spoons	141	64.1
≥3 dessert spoons	79	35.9
Soft drinks (n = 232)		
≥5× per week	61	26.3
<5× per week	171	73.7
Processed fruit juices (n =231)		
≥5× per week	76	32.9
<5× per week	155	67.1
Breakfast place (n = 230)		
Home	187	81.3
Outside	15	6.5
No breakfast	28	12.2
Lunch place (n = 224)		
Home	200	89.3
Outside	20	8.9
No lunch	4	1.8
Dining place (n = 232)		
Home	214	92.2
Outside	5	2.2
No dinner	13	5.6

FV = Fruits and Vegetables.

**Table 3:** Anthropometric and clinical characteristics by frequency of bean consumption of adolescents. Niterói—RJ, 2006–2007.

	General	Up to 4× Weekly	5 a 6× Weekly Up to 1× Daily	2× or Plus Daily
**BMI**	20.68	21.54 *	20.37	20.56
	(18.89–24.33)	(19.77–26.76)	(18.78–24.53)	(18.74–23.27)
**% BF**	23.20	27.05 ^§^	25.25	25.00
	(17.55–29.00)	(22.15–33.22)	(23.00–27.32)	(16.50–28.20)
**WER**	42.14	45.60 *	42.24	41.86
	(39.94–46.58)	(40.98–48.62)	(39.66–45.99)	(39.62–46.02)
**WC**	69.67	70.33	69.17	69.67
	(64.67–75.67)	(65.37–78.83)	(64.58–74.25)	(64.67–75.50)
**ABC**	75.33	77.58	76.08	74.83
	(69.33–82.23)	(71.41–85.33)	(69.63–83.92)	(68.50–82.00)
**WHR**	0.77	0.75	0.77	0.77
	(0.73–0.80)	(0.72–0.80)	(0.72–0.80)	(0.74–0.80)
**TST**	16.83	19.33 *	17.92	15.53
	(10.88–23.37)	(14.58–26.58)	(12.58–23.18)	(10.30–22.87)
**DBP**	64.50	65.00	66.50	63.00
	(59.50–71.12)	(60.75–72.62)	(62.50–71.50)	(58.50–71.00)
**SBP**	110.25	110.50	110.50	110.00
	(101.87–116.12)	(101.87–115.50)	(102.00–118.25)	(101.00–117.00)
**Glucose**	90.83	90.62	88.11 *	91.77
	(84.96–97.37)	(84.73–97.64)	(80.94–92.74)	(86.79–98.36)
**HbA1c**	5.70	5.90	5.7	5.65
	(5.20–6.20)	(5.00–6.42)	(5.30–5.97)	(5.10–6.20)
**TG**	55.33	62.23	52.76	55.07
	(43.69–75.20)	(50.19–85.57)	(43.18–89.57)	(43.00–69.95)
**TC**	146.69	161.69 *^,+^	145.61	144.64
	(130.09–173.16)	(143.29–187.60)	(128.91–164.95)	(128.00–171.00)
**LDL-c**	84.55	97.32 *^,+^	82.71	83.37
	(70.10–103.28)	(79.94–124.25)	(67.71–104.14)	(67.53–100.97)
**Non HDL-c**	96.73	109.87 *^,+^	95.88	96.00
	(80.44–117.91)	(91.43–136.75)	(80.99–118.26)	(78.00–114.95)
**HDL-c**	49.20	51.82	48.95	48.01
	(42.10–56.76)	(44.28–56.64)	(43.43–54.40)	(42.80–57.29)

* *p* < 0.05 compared to the consumption category 2 or more times daily; ^§^ *p* ≤ 0.001 compared to consumption category 2 or more times daily; ^+^ *p* < 0.05 compared to the consumption category 5 to 6 times per week up to 1 × daily. BMI (Kg/m^2^): body mass index, n = 231; %BF: body fat percentage, n = 229; WER: waist to height ratio, n = 231; WC (cm): waist circumference, n = 231; ABC: abdominal circumference (cm), n = 231; WHR: waist-hip ratio, n = 231; TST (mm): tricipital skinfold thickness, n = 230; DBP (mmHg): diastolic blood pressure, n = 230; SBP (mmHg): systolic blood pressure, n = 230; Gly (mg/dL): glucose, n = 212; HbA1c (%): glycated haemoglobin, n = 214; TC (mg/dL): total cholesterol, n = 213; TG (mg/dL): triglycerides, n = 212; Non-HDL-c (mg/dL): CT minus HDL-c, n = 213; LDL-c (mg/dL): LDL cholesterol, n = 206; HDL-c (mg/dL): HDL cholesterol, n = 213. Data expressed as median (25th–75th percentiles).

**Table 4:** Independent factors for nutritional status outcomes of adolescents after controlling for confounding factors in multiple linear regression analysis. Niterói—RJ. 2006–2007.

BMI Model ^†^	β	SE	*p*
Bean consumption (reference: up to 4× weekly)			
5–6× per week up to 1× daily	−0.004	0.002	0.092
2× or plus daily	−0.004	0.002	0.030
Marital status (reference: single)			
Married	0.009	0.005	0.069
FV Frequency (reference: ≥5× day)			
<5× per day	−0.002	0.001	0.173
Lunch place (reference: at home)			
Outside	0.004	0.002	0.043
No lunch	0.013	0.005	0.010
Two-way interaction:			
Sexual maturation × energy consumption	−0.000	0.000	0.017
Physical activity × energy consumption	−0.000	0.000	0.048
**% Body Fat Model**	**β**	**SE**	***p***
Bean consumption (reference: up to 4× weekly)			
5–6× per week up to 1× daily	−3.842	1.743	0.029
2× or plus daily	−3.372	1.482	0.024
Marital status (reference: single)			
Married	7.811	3.641	0.034
Alcohol consumption (reference: no)			
Yes	−2.301	1.791	0.200
Lunch place (reference: at home)			
Outside	−1.310	1.636	0.424
No	11.277	3.812	0.004
Two-way interaction:			
Gender × age	−1.194	0.471	0.012
**LDL-c Model**	**β**	**SE**	***p***
Bean consumption (reference: up to 4× weekly)			
5–6× per week up to 1× daily	−0.187	0.073	0.012
2× or plus daily	−0.176	0.064	0.007
Per-capita income (reference: up to R$ 200.00)			
>R$200.00 up to R$400.00	−0.108	0.053	0.042
>R$400.00	−0.002	0.069	0.978
Sedentary activities (reference: <2 h/day)			
≥2 h/day	−0.094	0.061	0.123
Frequency of fruit consumption (reference: ≥3× day)			
<3× day	−0.122	0.064	0.059
Breakfast (reference: yes)			
No	0.219	0.082	0.009
Soft drinks consumption (reference: ≥5× weekly)			
<5× weekly	−0.095	0.054	0.080
Processed juices consumption (reference: ≥5× weekly)			
<5× weekly	−0.116	0.056	0.040
Two-way interaction:			
Physical activity × energy consumption	−0.000	0.000	0.039

β = effect estimate; SE = standard error; BMI = body mass index; % Body Fat = body fat percentage; LDL-c = LDL cholesterol; FV = fruits and vegetables. **^†^** Adjusted for gender, age, menarche/axillary pilosity, physical activity and energy consumption.

**Table 5:** Energy consumption according to level of physical activity of adolescents. Niterói—RJ, 2006–2007.

	Physical Activity (Minutes/Week)		*p*
	≥300	<300	
**Total (Kcal)**	2936.24	2533.81	0.112
	(2535.26–3804.73)	(1964.16–3200.68)	
**FV (Kcal)**	63.87	82.72	0.050
	(38.94–94.54)	(52.23–121.84)	
**Culinary preparation (%)**	34.16	38.60	0.035
	(28.25–41.67)	(31.03–45.19)	
**Processed and Ultra-processed (%)**	65.84	61.40	0.035
	(58.33–71.75)	(54.81–68.97)	

Data expressed as median (25th–75th percentiles). FV = fruits and vegetables.

## Abbreviations

BMI, Body Mass Index; Camelia, Cardio-metabólico-renal e familiar; ERICA, Study of Cardiovascular Risks in Adolescents; FDP, Family Doctors Programme; FFSQ, Food Frequency Semiquantitative Questionnaire; FV, Fruits e Vegetables; Gli, Glucose; HbA1c, Glycated haemoglobin; HDL-c, HDL-cholesterol; HUAP, Antonio Pedro University Hospital; LDL-c, LDL-cholesterol; PENSE, National School Health Survey; % BF, Body Fat percentage; POF, Family Budget Survey; TC, Total Cholesterol; TG, Triglycerides; TST, Tricipital Skin Fold; WER, Waist-height Ratio; WHR, Waist-hip ratio; UFF, Fluminense Federal University.
